# Supplementary material for: From sequence to enzyme mechanism using multi-label machine learning
Source: BMC Bioinformatics. 2014 May 19;15:150. doi: 10.1186/1471-2105-15-150 (PMC4229970; doi:10.1186/1471-2105-15-150)
Supplement: Additional file 2 — Java code of ml2db. Additional file ml2db_code.tar.gz contains the Java source code to run the multi-label machine learning experiments and save the results to database. The code’s Javadoc is included. [file 1471-2105-15-150-S2.zip › additional file 2/ml2db/utils/doc/cern/jet/random/engine/package-tree.html]

cern.jet.random.engine Class Hierarchy


JavaScript is disabled on your browser.


- Overview
- Package
- Class
- Use
- Tree
- Deprecated
- Index
- Help

- Prev
- Next

- Frames
- No Frames

- All Classes

# Hierarchy For Package cern.jet.random.engine

Package Hierarchies:

- All Packages

## Class Hierarchy

- java.lang.Object
  - cern.colt.PersistentObject (implements java.lang.Cloneable, java.io.Serializable)
    - edu.cornell.lassp.houle.RngPack.RandomElement (implements java.lang.Cloneable)
      - edu.cornell.lassp.houle.RngPack.RandomSeedable
        - cern.jet.random.engine.RandomEngine (implements cern.colt.function.DoubleFunction, cern.colt.function.IntFunction)
          - cern.jet.random.engine.MersenneTwister

- Overview
- Package
- Class
- Use
- Tree
- Deprecated
- Index
- Help

- Prev
- Next

- Frames
- No Frames

- All Classes
